# Utility of contrast-enhanced magnetic resonance imaging for planning of surgical procedure in Paget’s disease of the breast

**DOI:** 10.1007/s00595-025-03016-y

**Published:** 2025-02-28

**Authors:** Takaaki Oba, Kazuma Maeno, Ryoko Iji, Nami Kiyosawa, Yonghan Park, Hiroki Morikawa, Masatsugu Amitani, Tadafumi Shimizu, Mayu Ono, Tokiko Ito, Toshiharu Kanai, Hisanori Matoba, Fumiyoshi Takayama, Ken-ichi Ito

**Affiliations:** 1 Division of Breast and Endocrine Surgery, Department of Surgery, Shinshu University School of Medicine, 3-1-1 Asahi, Matsumoto, Nagano, 390-8621 Japan; 2 Department of Surgery, Shinshu University School of Medicine, Matsumoto, Japan; 3 Department of Infection and Host Defense, Shinshu University School of Medicine, Matsumoto, Japan; 4 Department of Molecular Pathology, Shinshu University School of Medicine, Matsumoto, Japan; 5 Department of Radiology, Chikuma Central Hospital, Nagano, Japan

**Keywords:** Paget’s disease, Breast conserving surgery, Magnetic resonance imaging

## Abstract

**Purpose:**

Contrast-enhanced magnetic resonance imaging (CE-MRI) is an essential imaging modality for planning breast cancer surgical procedures. However, CE-MRI findings in Paget’s disease of the breast (PD) have not been studied extensively. This study aimed to elucidate the CE-MRI findings of PD and assess their role in surgical treatment planning.

**Methods:**

Twelve patients with PD who underwent surgery between 2011 and 2023 were retrospectively analyzed.

**Results:**

The average patient age was 73.8 ± 14.3 years. CE-MRI detected enhanced areas in the nipple-areola complex (NAC) and/or surrounding skin in all patients. Additionally, 6 patients showed enhanced areas within the breast, suggesting ductal spread into the breast. Of these, 1 patient underwent breast-conserving surgery (BCS), and 5 opted for mastectomy. Pathology confirmed the extent of ductal spread of PD, as indicated by CE-MRI. Among the 6 patients who did not have an enhanced area in the breast, 3 underwent BCS or central lumpectomy including NAC, and 3 chose mastectomy based on the patient's preference, and no malignant foci were observed in the breast.

**Conclusion:**

CE-MRI effectively evaluated the ductal spread of PD in the breast, demonstrating its utility in guiding the selection of surgical procedures.

**Supplementary Information:**

The online version contains supplementary material available at 10.1007/s00595-025-03016-y.

## Introduction

Paget’s disease of the breast (PD) is a rare malignancy originates from epithelial cells in the nipple and accounts for 0.5% of all breast cancers [1]. This disease has a unique characteristic: it widely spreads to the skin around the nipple-areola complex (NAC) and presents as erosion around the NAC. Additionally, PD can cause ductal spreading within the breast [1]. However, detecting the skin and ductal spread of PD with conventional imaging studies, including mammography and ultrasonography (US) [2–4]. Another characteristic feature of PD is the high incidence of multicentric or multifocal breast cancer [1]. Given the clinical characteristics of PD and the limitations of conventional imaging modalities, mastectomy has traditionally been the standard surgical procedure for treating PD [5].

In the preoperative imaging workup for breast cancer, contrast-enhanced magnetic resonance imaging (CE-MRI ), which provides detailed information on the extent of breast cancer, is essential to determine the surgical procedure (mastectomy or BCS). CE-MRI has proven useful in ductal carcinoma, the most common histological type of breast cancer, enabling the visualization of invasive lesions and intraductal spread [6, 7]. However, the potential of CE-MRI to detect the accompanying ductal spread in PD has not been well studied. Skin spread is also a critical determinant of the surgical procedure, and the extent of skin excision is usually determined on the basis of visible skin erosion. However, the ability of CE-MRI to estimate the extent of PD in skin erosion remains unknown.

This study aimed to evaluate the role of CE-MRI in determining the surgical procedure for patients with PD. We retrospectively analyzed the outcomes of 12 consecutive patients with PD who underwent CE-MRI before surgery, and examined the correlation between CE-MRI findings and physical and histopathological examination results.

## Methods

### Patients and study design

We retrospectively examined a cohort of 2052 patients with breast malignancies who underwent surgery at Shinshu University Hospital between January 2011 and September 2023. From these patients, those who underwent preoperative CE-MRI and who were diagnosed with PD based on histopathological examination of the resected specimens were selected. Twelve patients (0.6%) were included in this study.

### CE-MRI protocol

CE-MRI was performed preoperatively to assess the skin and ductal spread of PD and any additional underlying breast cancers. From January 2011 to April 2022, CE-MRI was performed using a 1.5 T Signa EXCITE HDxt system (GE Healthcare), equipped with a dedicated breast coil. From April 2022, we used the 3 T MAGNETOM Prisma System (SIEMENS). The whole breast on the affected side was imaged with the patient in prone position. Dynamic studies were performed before and 7 min after the intravenous administration of gadolinium-diethylenetriamine pentaacetate. The post-processing procedure included obtaining maximum-intensity projections.

A rapid initial enhancement with washout or a rapid-plateau enhancement pattern on NAC was considered as a PD lesion in the skin. These enhancement patterns in the breast that were continuous with NAC were interpreted as suggestive of ductal spread of PD.

### Surgical treatment

**Fig. 1:**
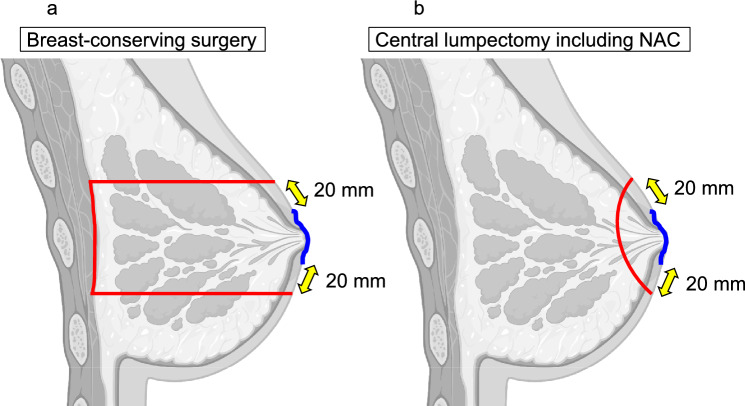
Surgical procedure for PD **a** Breast-conserving surgery removes the skin around the NAC with a margin of 20 mm of healthy skin. It also involves to remove breast tissue, including the NAC, vertically, down to the pectoralis major fascia. **b** Central lumpectomy, including the NAC removes the tumor and subareolar breast tissue, including the skin around the NAC with a margin of 20 mm of healthy skin. The resection line is indicated as red line in both procedures. The extent of the PD lesion is indicated by the blue line in both procedures. *PD* Paget’s disease of the breast, *NAC* nipple-areola complex

Mastectomy is recommended for patients in whom wide ductal spread of PD or other multifocal breast cancer is suspected based on CE-MRI findings. Patients assessed as having a limited extent of PD underwent BCS or central lumpectomy including NAC. BCS was defined as resection of the skin around the NAC with a margin of 20 mm of healthy skin and breast tissue, including the NAC vertically down to the pectoralis major fascia (Fig. 1a). Patients eligible for BCS also underwent mastectomy if they preferred it. Central lumpectomy, including NAC, involves resection of the tumor and subareolar breast tissue, including the skin around the NAC, with a margin of 20 mm of healthy skin (Fig. 1b). General anesthesia was chosen except for patients who were considered intolerant to general anesthesia because they were older or had comorbidities. Local anesthesia with 1% lidocaine was administered to patients who could not tolerate general anesthesia. Sentinel lymph node biopsy (SNB) was performed using the dual radioisotope and dye methods.

### Pathological diagnosis

The diagnosis of PD was confirmed via a histopathological examination. Breast tissue samples, including NAC, were fixed in 10% buffered formalin for 24–48 h and subsequently embedded in paraffin. Serial Sects. (3 µm in thickness) were cut from the paraffin blocks and stained with hematoxylin and eosin (HE). The microscopic assessment was performed by a pathologist (H.M.). PD was diagnosed based on the identification of characteristic large, pale-stained Paget cells within the epidermis, exhibiting abundant cytoplasm and prominent nucleoli. Immunohistochemical (IHC) staining of estrogen receptors (ERs) and progesterone receptors (PgRs) was performed to evaluate the hormone receptor status.

### Adjuvant therapy

Whole-breast irradiation at 50 Gy is recommended for patients undergoing BCS or central lumpectomy, including NAC. Breast reconstruction was performed according to patient preference for patients who underwent mastectomy. Patients positive for ER or PgR in PD or other underlying breast cancers were treated with tamoxifen 5 years after surgery.

### Data collection

Clinical information, including age, sex, estimated extent of PD skin spread by visual assessment, CE-MRI findings, surgical procedure, reasons for surgical choice, anesthesia type, extent of pathological skin and ductal spread of PD, ER and PgR status, and adjuvant therapy, were retrospectively collected from medical records.

### Ethics statement

This study complied with the provisions of the Declaration of Helsinki (64th WMA General Assembly, Fortaleza, Brazil; October, 2013). The local ethics committee also approved this study for clinical investigation at Shinshu University (no. 5350). Our institution uses a form on its website to enable patients to opt out of using their clinical data for research purposes. The requirement for written informed consent was waived. All patient data were anonymized.

## Results

### Patient characteristics

Table 1 presents the clinical characteristics of the patients included in this study are presented in Table 1. The mean age at the diagnosis was 73.8 ± 14.3 years. Of these, 91.7% (11 patients) were female, and 8.3% (one patient) were male. All patients exhibited clinical findings on NAC: 75.0% (9 patients) presented with eczema and 25.0% (3 patients) had erythema. None of the patients had any palpable breast masses. All patients were preoperatively diagnosed with PD using nipple-skin biopsy. The median follow-up period was 48 months (range: 3–129 months).

**Table 1:** Clinical presentations of 12 patients

No	Age	Sex	Chief complaints	Palpable mass in the breast	Abnormal findings in MMG	Abnormal findings in US at 1st visit
1	94	f	Eczema at NAC	None	None	None
2	89	f	Eczema at NAC	None	None	None
3	48	f	Erythema at NAC	None	None	None
4	69	f	Eczema on NAC	None	None	None
5	80	f	Eczema on NAC	None	None	None
6	81	m	Erythema on NAC	None	None	None
7	56	f	Eczema on NAC	None	None	None
8	76	f	Erythema on NAC	None	None	None
9	77	f	Eczema on NAC	None	None	None
10	82	f	Eczema on NAC	None	None	None
11	79	f	Eczema on NAC	None	None	None
12	54	f	Eczema on NAC	None	None	None

*MMG* mammography, *US* ultrasonography, *NAC* nipple-areola complex

### Preoperative imaging workup

All patients underwent preoperative imaging examinations using mammography, ultrasonography, and CE-MRI. None of the patients had abnormal mammographic findings. Additionally, US revealed no abnormal findings in the breast during the first examination in any patient (Table 1). Patients 4 and 10 underwent 3 T MRI, while the others underwent 1.5 T MRI.

**Fig. 2:**
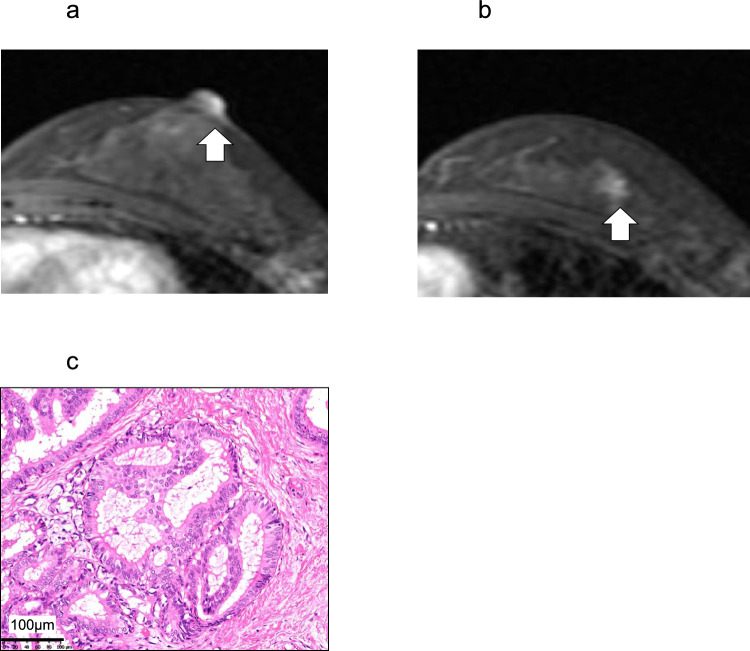
CE-MRI and histopathological (HE stain × 200) findings of Patient 12. **a** CE-MRI showing an enhancement area of 5 mm (arrow) on the NAC diagnosed as limited PD. **b** Another enhancement area in the upper outer quadrant of the breast is detected (arrow). **c** The enhancement area shown in **b** was diagnosed as DCIS. Scale bar = 100 μm. *CE-MRI* contrast-enhanced magnetic resonance imaging, *HE* hematoxylin and eosin, *NAC* nipple-areola complex, *PD* Paget’s disease of the breast, *DCIS* ductal carcinoma *in situ*

**Fig. 3:**
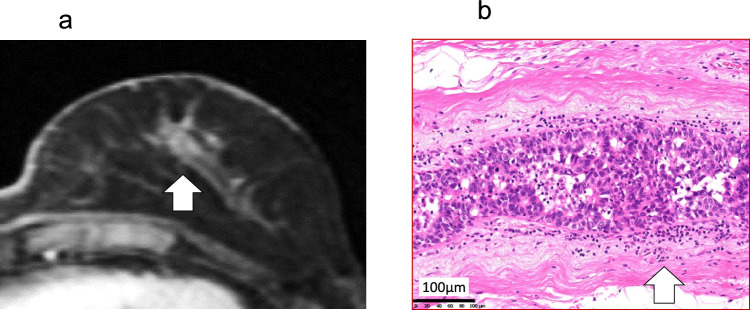
CE-MRI, and histopathological (HE stain × 200) findings of Patient 11. **a** CE-MRI shows a segmental enhancement area in the upper section of the breast, which is continuous with the enhancement area on the NAC (arrow). **b** The enhanced area in the breast was diagnosed as a ductal component of PD. Infiltration of inflammatory cells (arrow) in the stroma around the ductal component of PD. Scale bar = 100 μm. *CE-MRI* contrast-enhanced magnetic resonance imaging, *HE* hematoxylin and eosin, *NAC* nipple-areola complex, *PD* Paget’s disease of the breast

All patients had visible PD skin lesions on the NAC and adjacent skin, with erosive lesion sizes ranging from 5 to 65 mm. The extent of these lesions closely corresponded with the enhanced areas observed on CE-MRI; the maximum deviation was 7 mm in Patient 7 (Table 2). Half of the patients (6 patients) had additional enhanced lesions in the affected breast on CE-MRI, in addition to those on the NAC and surrounding skin. Of these, 1 patient (Patient 12) had another enhanced area in the upper outer quadrant of the affected breast unrelated to the PD lesion (Fig. 2a, b). This was confirmed to be ductal carcinoma in situ (DCIS) by core needle biopsy following second-look ultrasonography, which revealed a hypoechoic lesion consistent with the CE-MRI-enhanced area (Fig. 2c). The other 5 patients (Patients 4 and 8–11) showed enhanced areas contiguous with NAC, suggesting a possible ductal spread of PD (Fig. 3). However, no such spread was detected in these patients on second-look ultrasonography after CE-MRI.

**Table 2:** CE-MRI findings, surgical procedure, and histopathological findings

No	Visible skin lesion around NAC (mm)	CE-MRI		Surgical procedure			Reasons for choosing surgical procedure	Anesthesia	Histopathological findings		Receptor status	
		Enhancement area in skin around NAC (mm)	Enhancement area in breast (mm)	Breast	Axilla	Reconstruction			Extent of skin lesion (mm)	Extent of breast lesion (mm)	ER	PgR
1	15	18	None	Central lumpectomy including NAC	–	–	Limited extent	Local	17	–	1% >	0
2	10	10	None	Central lumpectomy including NAC	–	–	Limited extent	Local	12	–	0	0
3	25	30	None	BCS	SNB	–	Limited extent	General	26	–	0	0
4	5	5	18 (Continuous with NAC)	BCS	SNB	–	Limited extent	General	6	20 (Ductal spread of PD)	1% >	0
5	5	5	None	Mastectomy	SNB	–	Patient's preference	General	10	–	1% >	0
6	65	60	None	Mastectomy	SNB	–	Patient's preference	General	64	–	x	x
7	35	42	None	Mastectomy	SNB	+	Patient's preference	General	48	–	x	x
8	50	55	12 (Continuous with NAC)	Mastectomy	SNB	–	Patient's preference	General	45	12 (Ductal spread of PD)	60%	60%
9	5	5	10 (Continuous with NAC)	Mastectomy	SNB	+	Patient's preference	General	8	15 (Ductal spread of PD)	0	0
10	30	25	30 (Continuous with NAC)	Mastectomy	SNB	–	Wide ductal spread	General	25	30 (Ductal spread of PD)	0	0
11	40	38	42 (Continuous with NAC)	Mastectomy	SNB	–	Wide ductal spread	General	38	48 (Ductal spread of PD)	40%	0
12	5	5	15 (Not continuous with NAC)	Mastectomy	SNB	–	Another breast cancer	General	8	12 (Another DCIS)	0	0

*CE-MRI* contrast-enhanced magnetic resonance imaging, *NAC* nipple-areola complex, *BCS* breast-conserving surgery, *SNB* sentinel lymph node biopsy, *PD* Paget’s disease, *DCIS* ductal carcinoma in situ, *ER* estrogen receptor, *PgR* progesterone receptor

### Surgery

**Fig. 4:**
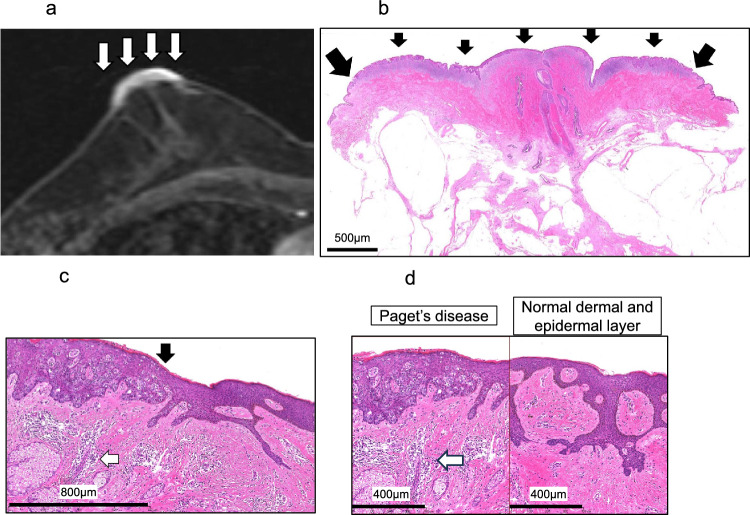
CE-MRI and histopathological findings of Patient 3. **a** CE-MRI shows an area of enhancement that is 30 mm in diameter (arrow) on the NAC with no evidence of ductal spread or other suspicious foci. **b** Histopathological findings (HE stain × 40) reveal PD extending 26 mm on the NAC (arrows showing the extent of PD), Scale bar = 500 μm. **c** The black arrow shows the histopathological border of PD and normal epidermis (HE staining × 100). The white arrow shows angiogenesis and infiltration of inflammatory cells in the dermis under PD. Scale bar = 800 μm. **d** Comparison of the histopathological findings (HE staining × 100) between PD and the normal dermal and epidermal layers. Angiogenesis and infiltration of inflammatory cells (white arrows) are observed in the dermis under PD. *CE-MRI* contrast-enhanced magnetic resonance imaging, *HE* hematoxylin and eosin, *NAC* nipple-areola complex, *PD* Paget’s disease of the breast

Table 2 outlines the surgical procedures and histopathological findings of resected specimens. BCS or central lumpectomy, including NAC, was performed in 25.0% of patients (Patients 1–4), while the remaining 75.0% (Patients 5–12) underwent mastectomy. Mastectomy was selected because of the suspected wide ductal spread of PD (Patients 10 and 11), the presence of another breast cancer (Patient 12), and patient preference (Patients 5–9). BCS or central lumpectomy, including NAC, was selected for Patients 1–3 in whom CE-MRI indicated no ductal spread of PD (Fig. 4a). For Patient 4, BCS was chosen because complete excision was deemed feasible despite the suspicion of ductal spread based on CE-MRI. Central lumpectomy, including NAC 1 and 2 was performed under local anesthesia in Patients 1 and 2, and these patients did not undergo SNB. The remaining ten patients (Patients 3–12) who underwent surgery under general anesthesia also underwent SNB. Two patients (Patients 7 and 9) underwent two-stage implant-based breast reconstruction.

### Histopathological findings

All 12 patients were histopathologically confirmed to have PD, consistent with the preoperative biopsy findings. Sentinel lymph node metastases were absent in all the 10 patients who underwent SNB. The histopathological extent of the skin spread, ranging from 8 to 64 mm, was almost congruent with visible lesions (Table 2). Furthermore, the extent of the histopathological spread of PD on the NAC or the surrounding skin closely corresponded to the CE-MRI estimates; the greatest discrepancy was 10 mm in Patient 8. The HE findings of patient 3 showed angiogenesis and infiltration of inflammatory cells in the dermis under the PD lesions, unlike the dermis under the normal epidermis (Fig. 4b–d).

In terms of the extent of PD within the breast, in the 3 patients who underwent BCS or central lumpectomy, including NAC (Patients 1–3), no ductal spread of PD was found on histopathological examination, which was concordant with the CE-MRI findings; hence, the margins in the breast were negative, and margins of more than 20 mm were achieved (Table 2). Conversely, the enhanced areas on CE-MRI continuous with NAC in Patients 4 and 8–11 were identified as ductal PD spread, with the pathological extent closely matching the CE-MRI estimates; the greatest discrepancy was 6 mm in Patient 11. Patient 4, who underwent BCS despite ductal spread, had a negative margin of > 20 mm. Furthermore, the histopathological extent of DCIS in Patient 12, which was unrelated to PD, corresponded to the CE-MRI enhancement area (Table 2). In the other 3 patients (Patients 5–7) treated with mastectomy and did not show an enhanced area in the breast on CE-MRI, malignant foci other than PD were not identified in the breast. ER was weakly positive in 4 patients (≥ 1% in Patients 1,4 and 5; 40% in Patient 11, and 60% in Patient 8), whereas PgR was positive in only 1 patient (60% for patient 7) (Table 2).

### Postoperative course

Three patients (25.0%) received adjuvant tamoxifen therapy (Patients 8, 11, and 12). Patients 8 and 11 were administered tamoxifen because of ER-positivity in their PD lesions. In Patient 12, tamoxifen was prescribed despite the PD being ER-negative, as the DCIS component was ER-positive. Patients 3 and 4 underwent whole-breast irradiation (50 Gy). Throughout the follow-up period, none of the patients experienced local recurrence or distant metastases (Table 3).

**Table 3:** Clinical course after surgery

No	Adjuvant therapy		Follow-up period (months)	Recurrence
	Endocrine therapy	Irradiation		
1	None	None	104	–
2	None	None	22	–
3	None	+ (whole breast 50 Gy)	129	–
4	None	+ (whole breast 50 Gy)	3	–
5	None	None	21	–
6	None	None	52	–
7	None	None	56	–
8	TAM	None	38	–
9	None	None	44	–
10	None	None	3	–
11	TAM	None	92	–
12	TAM	None	117	–

*TAM* tamoxifen

### The potential of CE-MRI to estimate the extent of the skin or ductal spread of PD

Table 4 summarizes the differences between the extent of skin or ductal spread of PD based on the histopathological and CE-MRI findings. The discrepancy was calculated as the extent of histopathological findings minus that of CE-MRI findings. The skin and ductal spread of PD were both underestimated by CE-MRI by up to 6 mm (Patient 7 for skin spread and Patient 11 for ductal spread). The mean difference was 0.6 ± 5.0 mm for skin spread and 2.6 ± 2.7 mm for ductal spread.

**Table 4:** Discrepancy between CE-MRI and histopathological findings in the skin or ductal spread of PD

No	Skin spread (mm)	Ductal spread (mm)
1	−1	NA
2	2	NA
3	−4	NA
4	1	2
5	5	NA
6	4	NA
7	6	NA
8	−10	0
9	3	5
10	0	0
11	0	6
12	3	NA
Mean ± SD	0.6 ± 5.0	2.6 ± 2.7

*CE-MRI* contrast-enhanced magnetic resonance imaging, *PD* Paget's disease of the breast, *NA* not applicable

## Discussion

The present study demonstrates that CE-MRI can accurately depict not only the extent of PD spread on the skin but also its ductal spread. Hence, information from CE-MRI is useful for determining the appropriate surgical procedure for PD.

Historically, mastectomy has been the recommended surgical procedure for treating PD [5]. However, BCS has recently been accepted as a proper surgical procedure in patients with PD to a limited extent [8]. Bijker et al*.* demonstrated a relatively low 5-year local recurrence rate of 5.2% after central lumpectomy, including NAC and irradiation, suggesting its feasibility for limited-extent PD [9]. Consistent with this report, Marshall et al*.* recommended local excision and subsequent breast irradiation as alternatives to mastectomy, particularly for patients with no palpable mass or abnormal mammographic density, with a 5-year local control rate of 91% [10]. Subsequent studies have highlighted the role of CE-MRI in the surgical planning of PD. Siponen et al*.* reported that BCS was performed in 18 (31.0%) of 58 patients with PD and that only 1 patient (5.6%) developed local recurrence. They suggested that CE-MRI might be beneficial when considering BCS. However, in their study, only 14 patients underwent CE-MRI, while 18 underwent BCS [5]. Similarly, Dominici et al. demonstrated the usefulness of CE-MRI in surgical planning of PD. However, in their study of 51 patients with PD, CE-MRI was performed in 27 (52.9%) patients, while 37 (72.5%) patients underwent BCS. Thus, it is possible that CE-MRI findings were not sufficiently used when BCS was chosen, and it was unclear whether CE-MRI was truly useful in determining the surgical procedure for PD based on the results of their study. To date, no reports have described the association between CE-MRI and histopathological findings in patients with PD. Our study fills this gap by providing detailed information on the association between CE-MRI findings and histopathological findings of PD. Although the clinical practice guidelines from the National Comprehensive Cancer Network for breast cancer recommend a preoperative imaging work-up using CE-MRI, the primary goal of this recommendation is to identify otherwise clinically occult breast cancer rather than to assess the extent of ductal spread or skin involvement in PD [11]. In this regard, the results of this study showed that CE-MRI accurately predicts the extent of spread on the skin and ductal spread of PD, providing novel insights into treatment strategies for PD. When CE-MRI does not suggest widespread ductal spread of PD, BCS or central lumpectomy, including NAC, may be sufficient. Conversely, mastectomy was preferred when CE-MRI indicated extensive ductal spread or mammographically occult independent breast cancer.

Central lumpectomy, including NAC, is less invasive than BCS and can be safely performed under local anesthesia with minimal invasion. In an aging society in Japan, there is an increasing demand for minimally invasive surgeries in elderly patients. In this regard, central lumpectomy, including NAC, as a less volumetric procedure may be preferable if the tumor can be completely resected. In line with this context, in PD of the breast, the possibility of axillary lymph node metastasis is extremely low because PD is a malignancy *in situ,* and omission of axillary surgery is acceptable. Indeed, in our patient cohort, none of the patients had metastases to sentinel lymph nodes. Thus, a complete cure can be achieved if complete tumor resection is achieved using central lumpectomy including NAC alone. Furthermore, patients with PD experience symptoms such as eczema during NAC, necessitating surgical intervention [12]. Hence, central lumpectomy, including NAC, under local anesthesia may be a viable and minimally invasive option, particularly in elderly patients with limited ductal spread of PD. This was evidenced in our cohort, in which patients of > 85 years of age underwent curative operations under local anesthesia guided by preoperative CE-MRI.

In the present study, CE-MRI accurately evaluated not only the ductal spread of PD but also skin spread, with a mean discrepancy of 0.6 mm between CE-MRI and histopathological findings. The mechanisms underlying skin lesion enhancement in PD on CE-MRI are not fully understood. Dissecting these mechanisms may offer new insights into PD pathology. Generally, the enhanced area of breast cancer on CE-MRI is attributed to angiogenesis and inflammatory cell infiltration into the stroma surrounding the ducts affected by cancer [13–15]. As shown in Fig. 3b and Fig. 4c, d, such histopathological changes were observed in both the stroma around the ductal spread of PD and the dermis beneath the PD lesions, potentially leading to skin enhancement on CE-MRI in patients with PD.

The present study was associated with several limitations. First, the small sample size (12 patients) limits the generalizability of our findings. More extensive studies are needed to validate the utility of CE-MRI in the surgical planning of PD. However, one strength of this study is that we reported a detailed association between CE-MRI findings and histopathological findings in PD in each case, in contrast to previous studies [5, 8–10]. This information enabled us to evaluate the role of CE-MRI in planning surgical procedures. Second, the retrospective nature of the study may have introduced selection bias, as only patients who underwent surgery and preoperative CE-MRI were included. These limitations highlight the need for more comprehensive studies to fully establish the role of CE-MRI in the management of PD. Furthermore, although 83.3% of the patients (10 of 12) included in this study were examined using 1.5 T MRI, 3 T MRI is becoming increasingly common. 3 T MRI provides images with higher resolution and enhanced tissue contrast, and hence may have the potential to improve diagnostic accuracy in PD by better detecting subtle ductal spread or skin involvement. With 3 T MRI, central lumpectomy, including NAC or BCS, with even smaller margins (< 20 mm) may be achievable. Future studies using 3 T MRI are warranted.

In conclusion, CE-MRI is useful for evaluating the extent of PD, including ductal and skin spread, around the NAC. Mastectomy should be considered when CE-MRI findings suggest extensive ductal spread of the PD. However, in patients with limited ductal spread, as predicted by CE-MRI, BCS, or central lumpectomy including NAC, it may be the preferred surgical option.

## Supplementary Information

Below is the link to the electronic supplementary material.

Supplementary file1 (PPTX 737 KB)

Supplementary file2 (DOCX 45 KB)

## References

[1] Sakorafas GH, Blanchard K, Sarr MG, Farley DR. Paget’s disease of the breast. Cancer Treat Rev. 2001;27:9–18. 11237774 10.1053/ctrv.2000.0203

[2] Ikeda DM, Helvie MA, Frank TS, Chapel KL, Andersson IT. Paget disease of the nipple: radiologic-pathologic correlation. Radiology. 1993;189:89–94. 8396786 10.1148/radiology.189.1.8396786

[3] Lim HS, Jeong SJ, Lee JS, Park MH, Kim JW, Shin SS, et al. Paget disease of the breast: mammographic, US, and MR imaging findings with pathologic correlation. Radiographics. 2011;31:1973–87. 22084182 10.1148/rg.317115070

[4] Zakaria S, Pantvaidya G, Ghosh K, Degnim AC. Paget’s disease of the breast: accuracy of preoperative assessment. Breast Cancer Res Treat. 2007;102:137–42. 17028984 10.1007/s10549-006-9329-2

[5] Siponen E, Hukkinen K, Heikkilä P, Joensuu H, Leidenius M. Surgical treatment in Paget’s disease of the breast. Am J Surg. 2010;200:241–6. 20678619 10.1016/j.amjsurg.2009.07.044

[6] Orel SG, Schnall MD. MR imaging of the breast for the detection, diagnosis, and staging of breast cancer. Radiology. 2001;220:13–30. 11425968 10.1148/radiology.220.1.r01jl3113

[7] Buchanan CL, Morris EA, Dorn PL, Borgen PI, Van Zee KJ. Utility of breast magnetic resonance imaging in patients with occult primary breast cancer. Ann Surg Oncol. 2005;12:1045–53. 16244803 10.1245/ASO.2005.03.520

[8] Dominici LS, Lester S, Liao GS, Guo L, Specht M, Smith BL, et al. Current surgical approach to Paget’s disease. Am J Surg. 2012;204:18–22. 22036205 10.1016/j.amjsurg.2011.07.010

[9] Bijker N, Rutgers EJ, Duchateau L, Peterse JL, Julien JP, Cataliotti L. Breast-conserving therapy for Paget disease of the nipple: a prospective European Organization for Research and Treatment of Cancer study of 61 patients. Cancer. 2001;91:472–7. 11169928 10.1002/1097-0142(20010201)91:3<472::aid-cncr1024>3.0.co;2-q

[10] Marshall JK, Griffith KA, Haffty BG, Solin LJ, Vicini FA, McCormick B, et al. Conservative management of Paget disease of the breast with radiotherapy: 10- and 15-year results. Cancer. 2003;97:2142–9. 12712465 10.1002/cncr.11337

[11] National Comprehensive Cancer Network. NCCN Clinical Practice Guidelines in Oncology: Breast Cancer. Version 4.2024. NCCN, 2024. https://www.nccn.org/professionals/physician_gls/pdf/breast.pdf . Accessed on 20 Aug 2024

[12] Sandoval-Leon AC, Drews-Elger K, Gomez-Fernandez CR, Yepes MM, Lippman ME. Paget’s disease of the nipple. Breast Cancer Res Treat. 2013;141:1–12. 23929251 10.1007/s10549-013-2661-4

[13] Gilles R, Zafrani B, Guinebretière JM, Meunier M, Lucidarme O, Tardivon AA, et al. Ductal carcinoma *in situ*: MR imaging-histopathologic correlation. Radiology. 1995;196:415–9. 7617854 10.1148/radiology.196.2.7617854

[14] Heywang-Köbrunner SH, Hacker A, Sedlacek S. Magnetic resonance imaging: the evolution of breast imaging. Breast. 2013;22(Suppl 2):S77-82. 24074797 10.1016/j.breast.2013.07.014

[15] Oshida K, Nagashima T, Ueda T, Yagata H, Tanabe N, Nakano S, et al. Pharmacokinetic analysis of ductal carcinoma *in situ* of the breast using dynamic MR mammography. Eur Radiol. 2005;15:1353–60. 15789211 10.1007/s00330-005-2661-9

