# Supplementary material for: Utility of contrast-enhanced magnetic resonance imaging for planning of surgical procedure in Paget’s disease of the breast
Source: Surg Today. 2025 Feb 28;55(6):778–86. doi: 10.1007/s00595-025-03016-y (PMC12098499; doi:10.1007/s00595-025-03016-y)
Supplement: Supplementary file 1 — Supplementary file1 (PPTX 737 KB) [file 595_2025_3016_MOESM1_ESM.pptx]

## Slide 1
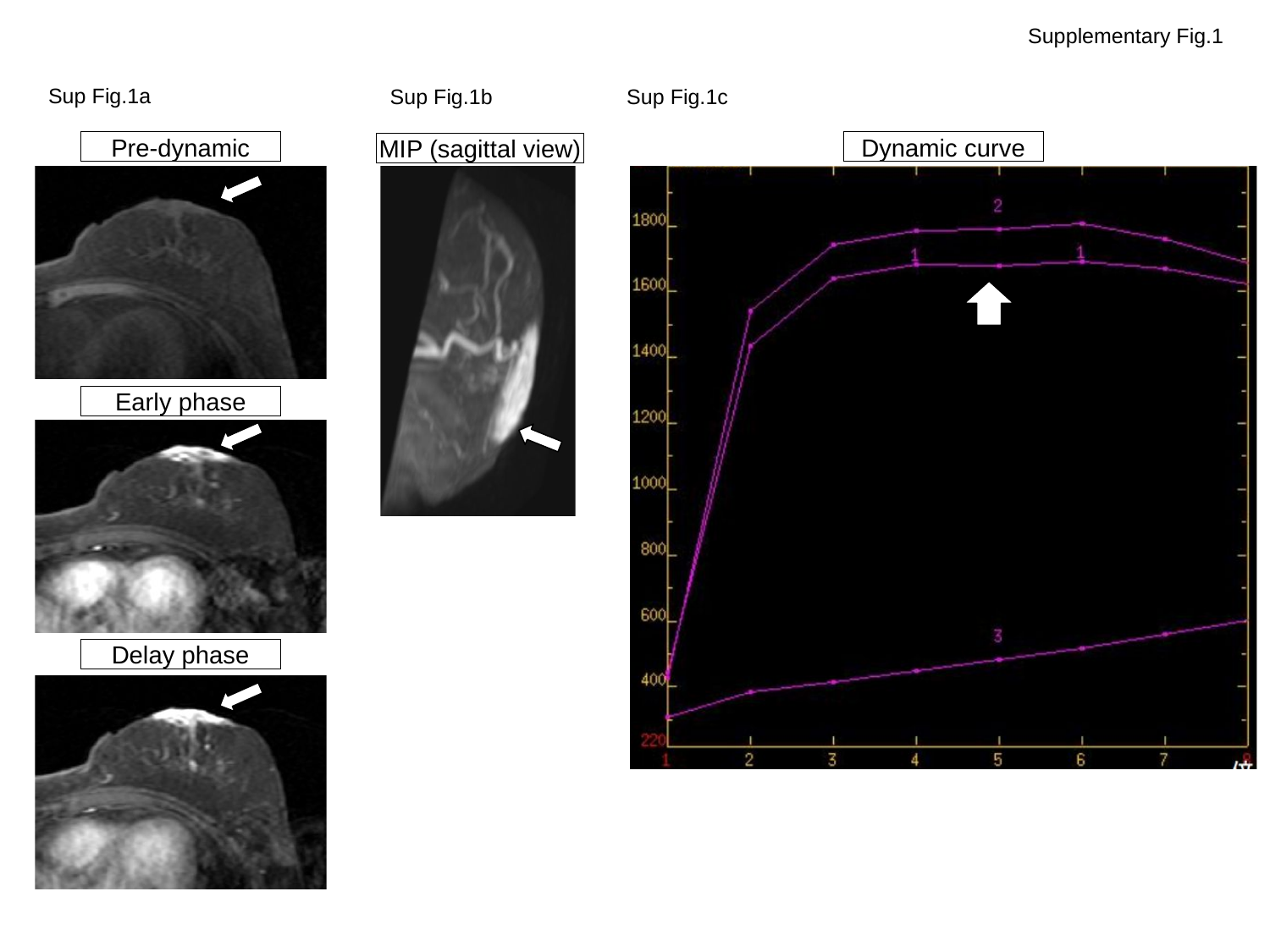

Supplementary Fig.1
Sup Fig.1a
Sup Fig.1b
Sup Fig.1c
Pre-dynamic
Dynamic curve
MIP (sagittal view)
Early phase
Delay phase
