# Supplementary material for: Utility of contrast-enhanced magnetic resonance imaging for planning of surgical procedure in Paget’s disease of the breast
Source: Surg Today. 2025 Feb 28;55(6):778–86. doi: 10.1007/s00595-025-03016-y (PMC12098499; doi:10.1007/s00595-025-03016-y)
Supplement: Supplementary file 2 — Supplementary file2 (DOCX 45 KB) [file 595_2025_3016_MOESM2_ESM.docx]

**Supplementary Figure Legends**

**Supplementary Figure 1. Dynamic study on CE-MRI of Patient 3**

(a) Pre-dynamic, early phase (1 min after injection of gadolinium-diethylenetriamine pentaacetate), and delay phase (5 mins after injection of gadolinium-diethylenetriamine pentaacetate) shows a rapid-plateau enhancement pattern on the NAC (arrow). (b) Maximum-intensity projections (MIP) (sagittal view) show enhancement area on the NAC (arrow) with no evidence of ductal spread or other malignant foci. (c) Dynamic curve of enhancement area on the NAC (arrow) demonstrates a rapid-plateau enhancement pattern. (arrow).

CE-MRI: contrast-enhanced magnetic resonance imaging; NAC: nipple-areola complex
